# Mental Health Gap Action Programme intervention Guide (mhGAP-IG) for Child and Adolescent Mental Health in Low- and Middle-Income Countries (LMIC): A Systematic Review

**DOI:** 10.1007/s10597-022-00981-3

**Published:** 2022-05-17

**Authors:** Marta Petagna, Charles Marley, Cristóbal Guerra, Clara Calia, Corinne Reid

**Affiliations:** 1 School of Health in Social Sciences, The University of Edinburgh, Edinburgh, UK; 2 School of Allied Health Science and Practice, The University of Adelaide, Engineering & Mathematical Sciences Building, North Terrace, 5001 Adelaide, Australia; 3 Universidad Santo Tomás, Santiago, Chile; 4 Victoria University, Melbourne, Australia

**Keywords:** Global Mental Health, mhGAP, mhGAP-IG, Child and Adolescent Mental Health, LMIC

## Abstract

**Background:**

The Mental Health Gap Action Programme (mhGAP) supports engagement of non-specialists in mental health services in Low- and Middle-Income countries. Given this aim, assessment of the effectiveness of approaches under its remit is warranted.

**Aims:**

We evaluated mhGAP approaches relating to child and adolescent mental health, focusing on provider / child outcomes, and barriers / facilitators of implementation.

**Methods:**

Thirteen databases were searched for reviews and primary research on mhGAP roll out for child and adolescent mental health.

**Results:**

Twelve studies were reviewed. Provider-level outcomes were restricted to knowledge gains, with limited evidence of other effects. Child-level outcomes included improved access to care, enhanced functioning and socio-emotional well-being. Organisational factors, clients and providers? attitudes and expectations, and transcultural considerations were barriers.

**Conclusions:**

Further attention to the practical and methodological aspects of implementation of evaluation may improve the quality of evidence of the effectiveness of approaches under its remit.

## Introduction

From the early 2000s, the Movement for Global Mental Health (GMH) has been advocating for the inclusion of Mental Health as a core component of the Global Health agenda, under the slogan *No Health Without Mental Health* (Prince et al., 2007). The 2007 Lancet series *Global Mental Health* (Lancet GMH Group, 2007) called for addressing the unethical gap between the population in need of mental health intervention and those who can access care (Lancet GMH Group, 2007). This gap was seen as particularly pronounced in low- and middle-income countries (LMICs) where social determinants of poor mental health have higher prevalence, formal mental health care systems are less well-structured, and specialised service providers are in short supply (Lancet GMH Group, 2007; Patel 2010; Whitely, 2015).

The Mental Health Gap Action Programme (mhGAP) was launched in 2008 (World Health Organisation [WHO], 2008b) to support scaling up services in LMIC. Reflecting a health-systems strengthening approach, the primary focus was the mainstreaming of mental health within primary health care services (WHO, 2008a). In 2010, the mhGAP Intervention Guide (mhGAP-IG) was released to provide non-specialised service providers with clinical algorithms to support the diagnosis and management of prioritised mental disorders, including two child related modules: ‘Developmental Disorders’ and ‘Behavioral Disorders’ (WHO, 2010). Subsequent additions broaden the spectrum of mental health difficulties to be managed in non-specialised settings (WHO, 2013, 2015). The most recent version, released in 2016, amongst other modifications, merged both child modules into the single category of ‘Child and Adolescent Mental and Behavioral Disorders’ (WHO, 2016). As of 2018, mhGAP-IG had been rolled out in over 100 countries and has been translated in more than 20 languages (WHO,^2018^).

Despite this growth, the approach has attracted criticism along cultural, ontological, and epistemological lines. Due to epistemological assumptions regarding how mental states can be identified and measured (Summerfield, 2008, 2012), it has been argued that it exports a western-based bio-medical ontology of mental health and illness (Bracken et al., 2016). Additionally, critics also argue that mental health and illness cannot be understood separately from the social and cultural context in which they originate (Kohrt & Griffith, 2015), thus questioning the generalisability of the diagnoses underpinning the approach. The empirical basis of the approach has also attracted criticism, with critics arguing that many western-based psychological and psychiatric treatments lack evidence of effectiveness and are potentially inferior to traditional and indigenous forms of healing (Fernando, 2014).

In response, advocates maintain that states of mental suffering manifest themselves in largely similar ways across cultures (Patel & Thornicroft, 2009) and that the GMH approach is open to including indigenous systems of meaning and healing (Patel, 2014). Advocates also argue that failure to address the ‘treatment gap’ based on cultural relativism amounts to denial of the basic rights to health and health care, thus contributing to the human rights violations those experience mental illness are subject to in indigenous and western societies alike (Patel, 2014). Prompted by these ethical imperatives, advocates argue it is necessary to bracket ontological and aetiological concerns (Bemme & D’Sousa, 2014) and concentrate efforts on providing access to treatments that have evidence of effectiveness (Whitley, 2015). Therefore, whilst the approach’s most immediate goal is to increase access to services (WHO, 2008b), its legitimacy is largely predicated on its approaches being effective at reducing the disabling effects considered to result from mental health disorders (WHO, 2018).

This argument acquires relevance when childhood mental disorders are discussed. Advocates for the approach maintain that in no area is the treatment gap as acute as it is in child and adolescent mental health (Kieling et al., 2011; Patel et al., 2008) and that the area should be prioritised for scaling up services (Patel & Rahman, 2015; Servili, 2012). As a counter, critics argue that the very notion of childhood underpinning the approach is culturally specific (Pupavac, 2011) and that its translation to other sociocultural milieus is inadequate and potentially oppressive (Mills, 2014). However, whilst determining the nature and extent of mental distress in children remains a complex endeavour (Canino & Alegria, 2008), there appears to be some evidence, albeit of often limited quality (Kieling, 2011), that preventative and treatment interventions are consistent across multiple cultural contexts (Kieling, 2011; Patel, 2015).

Despite this, important questions remain regarding whether the mhGAP programme and its Intervention Guide are the most appropriate vehicles for guiding development in these clinical domains. A recent systematic review (Keynejad et al., 2018) of mhGAP-IG in LMICs – focused on adults rather than children – did not reach any firm conclusions about outcomes and effectiveness. Additionally, the methodological quality of the studies was not reviewed, which is an important omission given the importance advocates place on the need for approaches to be effective at treating mental health difficulties.

### Objectives of the Review

Our review aims to synthesise and assess the existing evidence related to the implementation of mhGAP-IG in child mental health promotion, prevention, and care settings in LMICs. Specifically, our review will investigate: (1) implementation outcomes at provider level – including mental health knowledge, attitudes, skills, competencies, and practices of care providers; (2) implementation outcomes at child level – however defined; and (3) barriers and facilitators to the implementation process.

## Methods

### Inclusion/Exclusion Criteria

#### Study Design

The study follows the parameters set out by PRISMA and the Cochrane GMH Collaboration to evaluating the evidence available. In terms of inclusion, the review adopts a pragmatic approach (Barbui et al., 2017), hence including any type of study design, document, or evaluation of implementation of the mhGAP-IG focused on child and adolescent mental health prevention, promotion, and / or intervention in LMICs. The study has not been published on Prospero; however, the database was searched for similar studies, with no historic or current studies focusing on mhGAP-IG in child mental health promotion, prevention, and / or care in LMICs. The only study of the mhGAP-IG focussed on adult mental health, highlighting the requirement for consideration of child mental health promotion, prevention, and care.

#### Population

The review includes any study focusing on the use of mhGAP-IG for a population under 18 years of age. Studies with a broader age focus were included only if implementation of the mhGAP package explicitly focused on CAMH modules. The geographic scope of the review is limited to LMICs as per World Bank / OECD classification (The World Bank, 2019). In line with the principles of the Cochrane GMH Collaboration (Barbui et al., 2017), the review includes any experience of implementation of the mhGAP-IG for a child population, irrespective of whether this occurred in a specialised, non-specialised, or traditional health promotion / health care setting.

#### Outcome Measures

Reviewed studies were required to report outcomes, qualitatively or quantitatively, in relation to either of the following two dimensions: (i) provider-level knowledge, attitudes and practices related to child and adolescent mental health, including skills in case identification and diagnosis, in any specialised, non-specialised or traditional setting; and (ii) mental health promotion, prevention and treatment outcomes in children and adolescents, in any setting. Additionally, to explore the broader question of what factors may support or hinder the mhGAP-IG in CAMH settings, our review also included: (iii) any study describing barriers and / or facilitators to the roll-out process. These three foci were extracted from the stated aims of the mhGAP approach (World Health Organisation, 2008b), specifically the goal of increasing access to prevention, promotion, and treatment (programme objective) by training providers on evidence-based care protocols (programme strategy).

#### Other Inclusion/Exclusion Criteria

The review covers the period from 2010 (date of publication of the mhGAP-IG) to April 2020. Studies conducted in countries where the mhGAP-IG was limited to adult populations, and/or where the roll out was not extended to the CAMH module/s, were not included in the review. Studies that did not assess implementation outcomes or analyse barriers and facilitators to the implementation process were also excluded.

### Search Strategy

The search was conducted in two stages. In stage one, an initial search on the DOPHER, Cochrane and DARE databases, aimed to identify whether other systematic reviews had been conducted on the same topic. In stage two, searches for primary evidence were conducted on EMBASE, MEDLINE, CINHAL Plus, Global Health, SCOPUS, PsychINFO, PsychArticles, PUBMed, PUBMed Central, and ProQuest.

Search terms were “mental health gap action programme” AND “child*” OR “young pe*”; “mental health gap action program” AND “child*” OR “young pe*”; “mhGAP” AND “child*” OR “young pe*”. Searches were conducted in English. In addition to database searches, the reference lists of the final batch of papers were searched for relevant studies.

### Search Overview

**Fig. 1:**
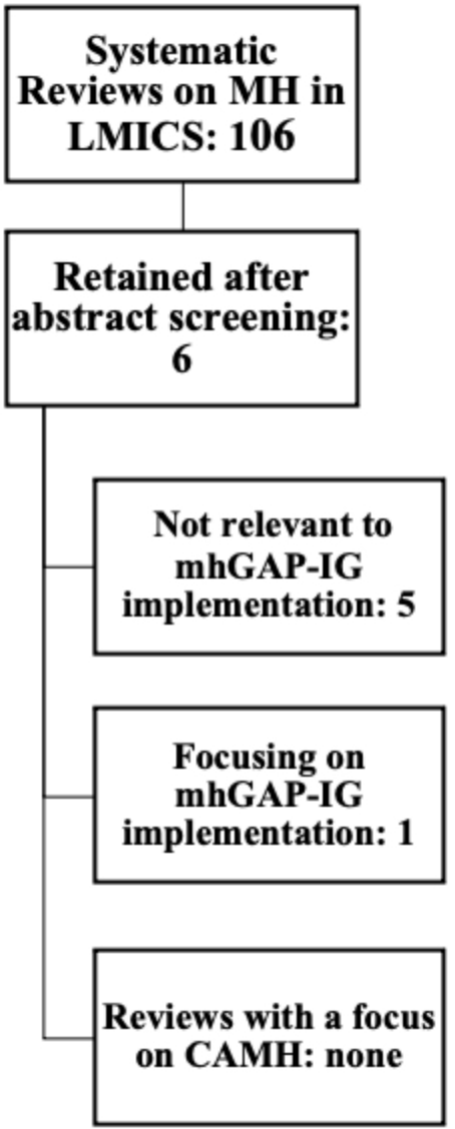
Search Results, Stage One

The initial search on DOPHER, Cochrane, and DARE databases did not retrieve any systematic reviews of the implementation of mhGAP-IG. By widening search criteria to a more generic “mental health” AND “low and middle income countries” OR “LMICs”, 106 studies were retrieved. After screening by keywords, titles, and abstracts, 100 reviews were eliminated. Of the remaining six, only one paper focussed on mhGAP-IG implementation (Keynejad et al., 2018), with no papers focusing on child and adolescent mental health. Details of the synthesis steps are outlined in Fig. 1.

The search for primary research articles in the remaining databases retrieved 895 papers; hand searches retrieved nine additional papers. After removing duplicates and screening titles/abstracts, 100 articles were retained for full text reading. Out of these, four were not accessible, and 48 did not have a focus on mhGAP-IG implementation.

Out of the remaining 48, 16 articles used quantitative, qualitative, or mixed method approaches to analyse the outcomes of mhGAP-IG training activities in terms of mental health knowledge, attitudes, and practices, including case identification skills. Five of these studies had a specific focus on CAMH. In two additional studies, CAMH modules were included in a broader mhGAP-IG training package.

In addition, 15 articles used quantitative or qualitative methodologies to analyse the outcomes of mhGAP-IG integration in terms of mental health promotion, prevention, and treatment outcomes. Four of these studies focused on CAMH, including one study protocol and one critical review, for which only the abstract was retrieved.

Finally, 17 articles illustrated the process of implementing mhGAP-IG approaches in specific countries or sets of countries. Whilst eight studies included an analysis of barriers and facilitators to the implementation process, none had a specific focus on CAMH, and one only was conducted in a setting where the implementation of mhGAP-IG approaches did not exclude children and adolescents.

**Fig. 2:**
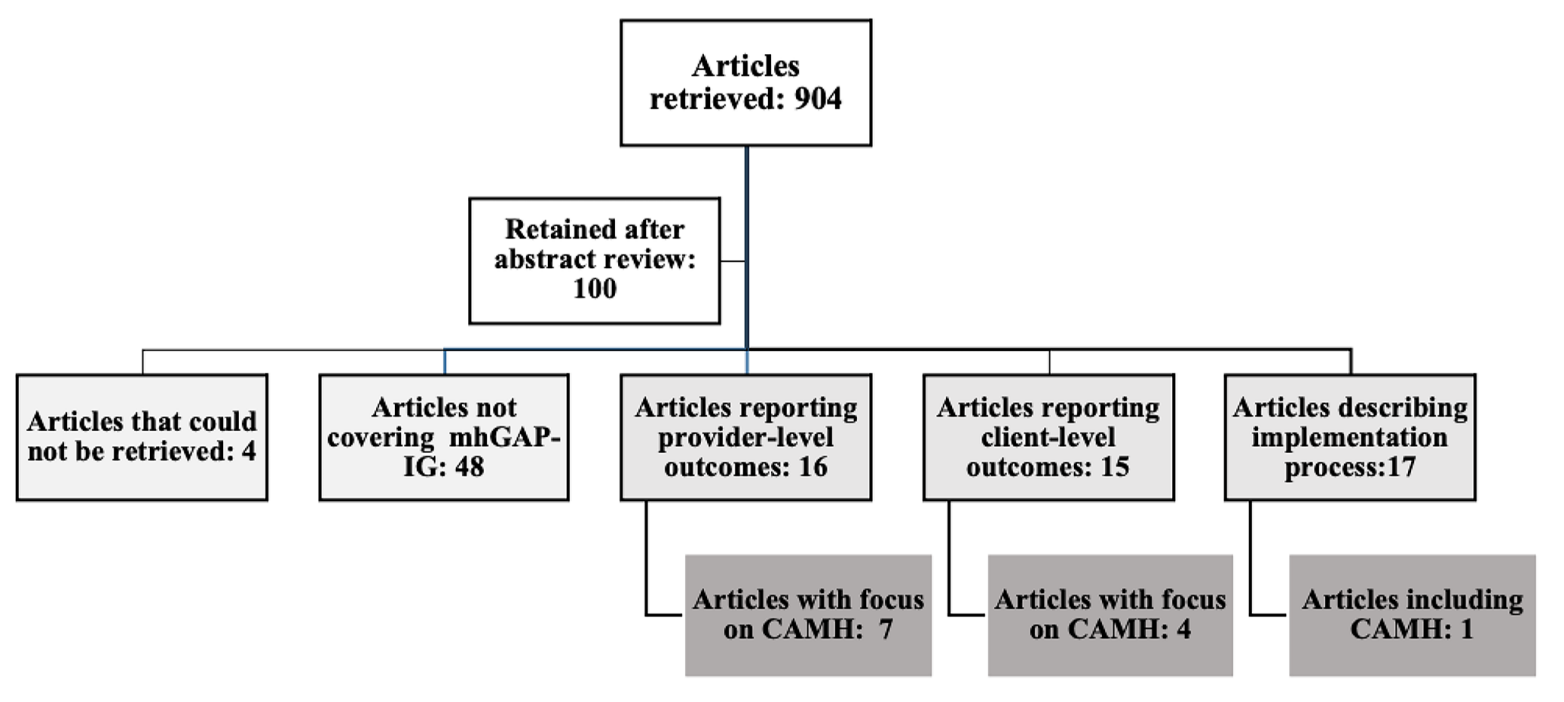
Search Results, Stage Two

The remainder of this paper will consider the articles that met the inclusion criteria set out above. Details of synthesis of primary research are outlined in Fig. 2.

### Quality Assessment

Given the broad nature of the inclusion criteria of our review, the final pool of studies selected were heterogeneous in objective, design, and methodological rigour. This is not unusual for reviews in public health, however, but it does highlight that a degree of flexibility is required for quality appraisal (Higgins et al., 2008).

Following the recommendations of the Cochrane Collaboration (Higgins et al., 2008), the criteria set out in the Quality Assessment Tool for Quantitative Studies (Effective Public Health Practice Project, 2008) was adopted as a guide. The following appraisal domains were prioritised for the assessment of studies focusing on provider- and client-level outcomes: study design; demographics; selection criteria; outcome measures; confounding variables; attrition; comprehensive reporting; and integrity of the intervention, including fidelity to the guidelines as well as local adaptations as required.

For the only paper within the ‘Barriers and Facilitators’ subset, quality assessment criteria were modified to reflect the focus on providers’ perspectives on potential implementation of mhGAP-IG approaches. Following recommendations from the Cochrane Collaboration Qualitative Methods Group (Hannes, 2011), the following dimensions were prioritised for the assessment: study design; demographics; selection criteria; methods of data collection and analysis; clear statement of findings; assessment of researcher’s influence on findings; justification of the conclusions.

## Results

### Characteristics of the Studies Included in the Review

The final pool of papers included in the review consisted of 12 studies. Seven explored outcomes at provider level. Of the seven, one study was conducted in a school setting (Lasisi et al., 2017), two focused on under- and post-graduate medical/health education (Murphy et al., 2017; Tesfaye et al., 2014), and the remaining four were conducted in rural primary health care facilities. Pooled together, the studies covered a sample of 1716 trainees, including 159 primary school teachers, 99 medical and health students, and 1,458 non-specialised health care providers. The remaining four studies (including one protocol) considered outcomes at the client level. All four focused on rural areas, with two conducted in clinical settings and two in community-based programmes. Pooled together, the studies covered a sample of 133 clients, in addition to 540 child-caregiver dyads targeted in the study protocol.

With regards to barriers and facilitators, only one study (Kane et al., 2016) met our criteria for review. The study explored providers’ views of anticipated challenges to the use of the mhGAP guidelines for the management of conditions related to stress (WHO, 2015), an annex to the core mhGAP-IG.

The literature search highlighted a key reason for the relative paucity of research regarding mhGAP-IG implementation for CAMH. A significant number of countries where mhGAP-IG was mainstreamed, the choice was made to only focus on adult mental health for integration – specifically, primary health care (PHC) only specific modules. This was the case for Uganda, Liberia, and Nepal (Kohrt et al., 2019); Tunisia (Spagnolo et al., 2017); Nigeria (Adebowale et al., 2014); South Africa (Sibeko et al., 2018); Kenya (Musyimi et al., 2018); Afghanistan (Khoja et al., 2016); Sri Lanka (Siriwardhana et al., 2013); Haiti (Mc Lean et al., 2015); Peru (Cavero, Diez-Canesco & Toyama, 2018), and others (Hanlon et al., 2016). This finding raises a concern –beyond the scope of this review – that in these countries, child and adolescent mental health care, in the absence of adequate protocols, may be informed by an adult mental health focus. Details of the study characteristics can be found in Table 1.

**Table 1:** Characteristics of the Studies Included in the Review

References	Location	Population	Study design	Outcome measures
Akol et al., (2018) ^a^	Uganda	Thirty-six PHCs	Randomised Controlled Trial	# or % of mhGAP-IG CAMH diagnoses
Akol. et al. (2017) ^b^	Uganda	Thirty-six PHC staff	Pre-post, uncontrolled cohort study	Contextualised standard mhGAP-IG knowledge test
Budosan et al., (2016) ^c^	Philippines	159 PHCs, 24 district and 8 provincial hospitals	Pre-post, uncontrolled cohort study	Non-standardised
Humayun et al., (2017) ^d^	Pakistan	58 staff and physicians	Pre-post, uncontrolled cohort study	Contextualised standard mhGAP-IG knowledge test
Lasisi et al., (2017) ^e^	Nigeria	159 primary school teachers	Non-randomised controlled trial	
Murphy et al. (2017) ^f^	UK and Somaliland	24 medical students	Mix-methods uncontrolled cohort study	a. Qualitative post-intervention feedback. b. ATP-30
Tesfaye et al., (2014) ^g^	Ethiopia	75 MSc students	Descriptive cohort study	Non-standardised
Alleyne (2017) ^h^	Barbados Island	Pediatric mental health service users	Abstract	Access to care
Grelotti et al. (2016) ^i^	Rural Haiti	65 patients	Retrospective cohort study	mhGAP-IG diagnoses
Hamdani et al., (2017) ^j^	Pakistan	540 child/ caregiver dyads	Two arm single-blind cluster randomised controlled trial	a. WHO DAS-Child b. Clinical Global Impressions; Strengths Difficulties Questionnaire; Pediatrics-Quality of Life, and others
Hamdani et al. (2015) ^k^	Pakistan	68 child/caregiver dyads	Uncontrolled pre-post cohort study	a. Standardised pre/post Knowledge, Attitudes, Practices questionnaire; b. WHO DAS-Child; c. Strengths and Difficulties Questionnaire d. Family Empowerment Scale f. WHO-5 Well-Being Index
Kane et al., (2016) ^l^	Uganda	19 mental health professionals	Qualitative study	a. Current practices for managing mental health problems; b. Barriers to the implementation of the guidelines; c. Possible solutions

PHC: Primary Health Care; mhGAP-IG: Mental Health Gap-Intervention Guide; CAMH: Child and Adolescent Mental Health; ATP-30: Attitudes Toward Psychiatry-30 item; WHO DAS-Child: The World Health Organization Disability Assessment Schedule for children; WHO-5: The World Health Organisation- Five Well-Being Index

### Summary of Quality Assessment of the Studies Included in the Review

The studies retrieved were methodologically heterogeneous, with over half appearing to be of mixed quality. At the provider level, only three (Akol et al., 2017, 2018; Lasisi et al., 2017) out of the seven studies had a clearly formulated research question. The others had loosely formulated evaluative objectives / aims (Humayun et al., 2017; Murphy et al., 2017; Budosan et al., 2016; Tesfaye et al., 2014). Only two studies (Akol et al., 2018; Lasisi et al., 2017) (one randomised: Akol et al., 2018) employed a control group. Four (Akol et al., 2017; Budosan et al., 2016; Humayun et al., 2017; Murphy et al., 2017) were designed as uncontrolled pre-post cohort studies. The seventh study (Tesfaye et al., 2014) was descriptive, with no baseline information provided. Four studies (Akol et al., 2017, 2018; Humayun et al., 2017; Lasisi et al., 2017) used standardised measures, two of which included the mhGAP-IG 25-items knowledge test (Akol et al., 2017; Humayun et al., 2017); the latter, however, was seen to only test ‘rudimentary knowledge’ of mental health (Akol et al., 2017). The other three studies (Budosan et al., 2016; Murphy et al., 2017; Tesfaye et al., 2014) used non-standardised measures. In three studies, participants were self-selected or selected by their supervisors (Akol et al., 2017; Lasisi et al., 20,017; Murphy et al., 2017). In three more, selection criteria were not mentioned (Budosan et al., 2016; Humayun et al., 2017; Tesfaye et al., 2014).

At the client level, one paper (Hamdani et al., 2017) provided a well-formulated protocol for a randomised controlled study. The other three papers included an uncontrolled, pre-post cohort study (Hamdani et al., 2015), a retrospective cohort study (Grelotti et al., 2016), and a critical review (Alleyne, 2017) for which no full text was retrieved. Only two studies (Hamdani et al., 2015; Hamdani et al., 2017) (including the protocol) had clearly formulated research questions and standardised measures. The study protocol (Grelotti et al., 2016) was the only paper with clearly defined selection criteria.

The study on barriers and facilitators (Kane et al., 2016) had well formulated research questions, providing a good description of the data collection methods and analytical approach, and a clear statement of findings. The study was less rigorous in the description of the study demographics, selection criteria, and in relation to consideration of the role the researchers in the construction of findings.

Overall, with a few exceptions, the literature retrieved appeared more preoccupied with documenting the experience of mhGAP-IG implementation than with rigorously evaluating it. Several studies claimed to demonstrate the ‘feasibility’ (Budosan et al., 2016; Grelotti et al., 2016; Hamdani et al., 2017) or ‘successful implementation’ (Tesfaye et al., 2014) of mhGAP-IG mainstreaming. Notably, however, feasibility and / or success were not clearly operationalised resulting in confusion and tautology, with the description of the implementation process offered as indicative of the demonstrated results. A complementary major limitation is that, in most of the cases Akol et al., 2017; Budosan et al., 2016; Humayun et al., 2017; Tesfaye et al., 2014; Hamdani et al., 2017; Hamdani et al., 2015), the authors were the same organisations/entities and people in charge of implementing the programme. This clearly introduced a risk of bias (Sanders, 2015) that the studies did not openly acknowledge, and for which no mitigation measures were reported. Table 2. summarises the findings of the quality assessment for the three sub-sets.

**Table 2:** Summary of Quality Assessment

	Study design	Selection criteria	Demographics	Confounding	Outcome measures	Attrition	Reporting	Integrity
**Provider-level**								
Akol et al., (2018)	2	2	2	1	1	n. a.	1	1
Akol et al., (2017)	2	1	2	0	1	1	2	2
Budosan et al., (2016)	0	0	1	0	0	0	0	1
Humayun et al., (2017)	1	0	1	0	1	0	2	2
Lasisi et al., (2017)	2	1	2	2	2	1	2	0
Murphy et al., (2017)	1	1	1	0	1	1	1	1
Tesfaye et al., (2014)	0	0	0	0	0	0	1	1
**Client-level**								
Alleyne (2017)	n. a.	n. a	n. a.	n. a.	n. a.	n. a.	n. a.	n. a.
Grelotti et al. (2015)	0	0	2	0	0	n. a.	0	n. a.
Hamdani et al., (2017)	2	2	2	n. a.	2	n. a.	n. a.	2
Hamdani et al. (2015)	2	0	2	0	2	0	1	2
**Barriers and facilitators**								
	**Study design**	**Selection criteria**	**Demographics**	**Researcher’s influence**	**Data collection/** **analysis**	**Statement of findings**	**Justification of conclusions**	
Kane et al., (2016)	2	1	1	1	2	2	2	

Ratings: 2 = well covered; 1 = adequately covered; 0 = poorly covered or not addressed.

### Summary of Provider-Level Outcomes

Whilst the mhGAP programme is conceptualised as an instrument for mental health prevention, promotion, and treatment (WHO, 2008b), the overarching aim of the studies assessing provider-level outcomes was narrowly formulated in terms of increasing providers’ ability to detect, identify, screen, diagnose, and manage child and adolescent mental health conditions. The ability to integrate developmental considerations in the assessment and management process, whilst included to some degree in the implementation guide (WHO, 2016), was not explicitly mentioned in the assessment of training results. Five studies reported that mhGAP-IG contents were adapted to the local context, and in some cases augmented with additional material, but none of the papers explicitly documented how local understandings of child mental health had been incorporated.

All seven papers in this sub-set concluded that the programme was effective in increasing non-specialists’ knowledge of child and adolescent mental health, generally defined as knowledge of “*symptoms, diagnosis, treatment, nature, causes and outcomes*” (Lasisi et al., 2017, p. 3) of categorically identified CAMH disorders. In three studies (Akol et al., 2017; Humayun et al., 2017; Lasisi et al., 2017), increases in knowledge were quantified and found to be statistically significant, with effect size only reported by Lasisi et al. (2017) and found to be large (Cohen’s *d* = 0.9). In the remaining four papers, knowledge gains were either self-reported (Budosan et al., 2016; Murphy et al., 2017) or assessed by course evaluators using non-standardised methods (Tesfaye et al., 2014) or inferred from other outcomes (e.g., increased diagnostic ability, as discussed below).

Humayun et al., (2017) found that, at baseline, CAMH was one of weakest knowledge areas for health care providers, with improvements reported – but not disaggregated – at post-test. Akol et al., (2017) found that, whilst physicians’ baseline knowledge was significantly higher than other personnel, cadre was not significantly associated with knowledge gains, supporting the conclusion that mhGAP-IG training may be equally suitable for all health professionals, regardless of qualification and profile. Murphy et al., (2017) suggested that technology can be leveraged to expand the applicability of mhGAP-IG, whilst Tesfaye et al., (2014) concluded that the contents of mhGAP-IG can usefully inform the development of academic curricula in CAMH – however, this statement needs to be qualified due the methodological limitations of the paper.

Effects of mhGAP-IG training on providers’ attitudes were only reported in two articles (Lasisi et al., 2017; Murphy et al., 2017), with the authors indicating that improvements were achieved in both cases. Lasisi et al., (2017) looked at teachers’ attitudes towards Attention-Deficit/Hyperactivity Disorder, reporting significant increases in positive views around school inclusion of students displaying behavioural difficulties, with moderate effect size (Cohen’s *d* = 0.5). Murphy et al., (2017) focused on generic attitudes towards psychiatry, reporting fewer stigmatising views towards the discipline but the improvement was only statistically significant (p = 0.011) for one of the two cohorts evaluated.

Effects on skills and practices were clearly reported in one study only. Akol et al., (2018) measured the impact of mhGAP-IG training on primary health care providers’ ability to diagnose CAMH conditions, obtaining non-significant results across primary health care services.

None of the studies within this sub-set attempted to measure other competencies, including the extent to which the psychosocial and broader environmental interventions recommended in mhGAP-IG were mainstreamed in the practice of non-specialised trainees. In addition, the studies did not consider whether this may be feasible at all in the context of brief facility-based primary health care consultations (Irving et al., 2017), which is important given the absence of coordinated and collaborative care across sectors in LMIC (Acharya et al., 2017; Budosan et al., 2016) found that even after successful training, most health care providers did not feel confident about providing psychosocial interventions, supporting Hamdani et al.’s view (2017) that mhGAP-IG training may provide health staff with knowledge about the *what* of mental health care, but not necessarily the skills that enable the *how* of service provision. Studies exploring mhGAP implementation for adult mental health have reached similar conclusions (Kohrt et al., 2019).

### Summary of Child-level Outcomes

At the child level, three dimensions of outcomes were analysed in the studies retrieved for this review: access to treatment (Alleyne, 2017), likelihood to receive a diagnosis in line with mhGAP classifications (Grelotti et al., 2016), and psychosocial functioning and emotional well-being of child and caregivers (Hamdani et al., 2015; Hamdani et al., 2017).

Alleyne (2017) compared three different models of CAMH service provision, all of which were reportedly conducive to increased access to care, but as the full text of this study was not retrieved, it was not possible to evaluate the strength of this conclusion. Grelotti et al. (2016) found that, within a mixed population of both children and adults affected by a humanitarian disaster, 75% received a diagnosis in line with mhGAP-IG classifications. Finally, Hamdani et al. (2015) found that mhGAP training of caregivers of children with intellectual disability or pervasive developmental disorders, augmented with mhGAP-recommended Parents Skills Training (PST: WHO, 2019), led to improved child and family functioning across a range of indicators and domains. Hamdani’s findings, whilst qualified in the light of the methodological limitations reported in our quality assessment, are in line with previous research evidence on task shifting approaches to supporting children with developmental disorders (Reichow et al., 2013), hence PTS being added to the battery of effective interventions for caregivers in community settings. A research protocol by the same authors (Hamdani et al., 2017) aims to replicate these results through a randomised controlled trial.

### Summary of Barriers and Facilitators

The literature search did not retrieve any studies focusing specifically on barriers and / or facilitators to implementing mhGAP-IG for child and adolescent mental health. This would appear to be significant research gap: since adult mental health components have been systematically prioritised in the programme’s roll out process at the expenses of the CAMH modules and dimensions, documenting the reasons for this exclusion would appear particularly important.

Only one study retrieved, whilst not having a specific focus on CAMH, explored barriers and facilitators where mhGAP’s CAMH modules were not excluded *a priori* (Kane et al., 2016). The study analysed the views of 19 mental health professionals working in primary health care services in rural Uganda. The barriers identified included high staff caseload, limited time per consultation, and mobility as impacting on clients’ ability to attend clinics, a key requirement of many psychosocial interventions. Similar obstacles were noted in the other two sub-sets of studies within this review. For example, Budosan et al., (2016) found that “lack of time” was a main factor hindering the provision of mhGAP-recommended packages of psychosocial care (p. 1170), with Akol et al., (2018) highlighting patient load, skill-mix, and clinic management as influencing programme outcomes.

With respect to the methodology of mhGAP-IG implementation, Kane et al.’s (2016) study indicated clients’ – and providers’ – preference for pharmacological treatment as a major barrier the prioritisation of psychosocial interventions as the first line of treatment for most conditions, an aim of the mhGAP approach (WHO, 2016). Other barriers include the perceived limited cultural appropriateness of some of the recommended psychosocial interventions, the requirement for adaptations to increase acceptability, as well as the role that socio-economic deprivation, social isolation, and poor physical health, play in affecting the long-term outcomes of psychological treatments (Kane et al., 2016). Whilst some of these observations were not made with specific reference to CAMH, most resonate within the context of this review, particularly the need to pair individual interventions to support children and families with ecological approaches to reducing risk factors in children’s environments.

## Discussion

Whilst critics of the GMH agenda have stimulated important discussions around GMH’s premises and goals, the importance of meeting the mental health needs of children and young people and enforcing the cessation of human rights violations against them (United Nations Human Rights Council, 2017) remains an urgent ethical imperative (Drew et al., 2011). The mhGAP may represent a useful instrument to achieve both results, however, findings from this review suggest that more robust evidence for CAMH at both the provider-, and child-level, is needed to support further investment in this tool.

At the provider level, whilst almost all the studies included in the review pointed to an increase in providers’ knowledge, none of them adequately explored the question of whether such knowledge-gains translate into increased competence and transformed practices. Recent studies focusing on mhGAP implementation for adult mental health have shown that the link between these three dimensions is feeble at best (Kohrt et al., 2019).

At the child level, whilst some interesting results were identified – i.e., better outcomes for children diagnosed with developmental disorders – the paucity of research, the heterogeneity of results, and methodological limitations of the studies from which the results were drawn, restrict the conclusions that can be drawn. Consequently, our first finding is that more, better, and independent intervention research is needed to support additional investments in the mhGAP programme for CAMH. Other studies have highlighted the dearth of good quality research evidence on CAMH interventions in LMICs (Klasen & Crombag, 2013) and, several years later, this appears to remain a significant gap (Babatunde et al., 2019). A related consideration is the need to document the reasons why the roll out of CAMH modules has often been excluded from the mhGAP-IG implementation process, and to investigate whether, in the absence of a specific focus on CAMH in many countries, clinical decision-making for child and adolescent mental health is informed by an adult mental health paradigm.

Secondly, the analysis of barriers and facilitators suggests that attention to the modalities of mhGAP implementation is required, including cultural relevance on the one side, and required systemic changes on the other. Whilst most of the studies reviewed stated that cultural adaptations were made to mhGAP-IG to fit within the local contexts, none of the papers explicitly discussed whether local understandings of childhood, mental health, and / or illness were incorporated. Parallels can be drawn with the literature on mhGAP for adult mental health, where cultural relevance is often referred to (Davies & Lund, 2017), but incorporation is often in response to community stigma against mental illness or an attempt to remove prejudice against accessing mental health care (Gwaikolo et al., 2017), This, arguably, creates the appearance that local systems of knowledge and practices are rarely seen as resources and potential allies towards the goal of greater mental and psychosocial well-being (Cooper, 2016) but instead as a means by which to increase acceptance of western approaches. Similarly, structural aspects of the existing health systems – including workforce size, workloads, and motivational factors, as well as client-provider hierarchies and dynamics – appear to play a fundamental role in facilitating or hindering the achievement of mhGAP goals (Gajaria et al., 2019; Kohrt et al., 2019; Mugisha et al., 2017; Spagnolo et al., 2018). Whilst several studies selected for this review refer to these aspects in the discussion of findings (Akol et al., 2017, 2018; Budosan et al., 2016), it is less clear whether these were systematically factored in the formulation of implementation strategies. The recently released *mhGAP Operational Manual* may contribute to addressing this gap but its impact is yet to be evaluated (WHO, 2018).

Thirdly, with the partial exception of Hamdani et al. (2015, 2017) and Humayun et al., (2017), the studies reviewed do not appear to reflect an ecological or developmental approach to CAMH. There was also no evidence of attempts to broaden the scope of mhGAP-IG implementation to include participatory mental health care planning, community mobilisation, and other complementary interventions recommended by the programme (Babalola et al., 2017), which characterises the mainstreaming of mhGAP approaches to adult mental health in some settings (Breuer et al., 2014; Kohrt et al., 2019). Despite schools having been identified as some of the most conducive environments for mental health prevention and promotion (Klasen & Crombag, 2013), in the studies selected for this review, only Lasisi et al. (2017) investigated implementation in an educational setting. Most of the studies were conducted in the context of facility-based service provision, suggesting an overall bias towards clinical approaches to child mental health care that is not in line with the inspiring principles of the mhGAP programme (Babalola et al., 2017; WHO, 2008b), or indeed of the GMH agenda more generally (Patel, 2014). And whilst the Intervention Guide recommends collaborative case management including family, school, and social welfare systems, the studies reviewed provide very limited examples of actual implementation of this model.

These last points lead to a final consideration, which is beyond the scope of this review: the extent to which the implementation of mhGAP reproduces western-based bio-medical ontology of mental health disorders as symptom-based categorical entities rather than the expression of a dynamic developmental process. By doing so, the important requirement to understand, interpret, and address child and adolescent mental health within an ecological understanding of child well-being (Skokauskas et al., 2019) is overlooked. The pragmatic approach to supporting clinical decision-making through simplified algorithms, which informs the Intervention Guide, may well be one of the reasons behind this lack of developmental focus. What was evident, however, was the limited consideration of a developmental focus across the studies included in this review, with the key preoccupation appearing to be the mainstreaming of knowledge and practices related to “*clinical presentations and treatment options*” (Akol et al., 2017, p. 4) and “*clinical features of psychiatric disorders*” (Tesfaye et al., 2014, p.3). The formulation of a specific Child-mhGAP Intervention Guide, as recently advocated (Skokauskas et al., 2019), may contribute to addressing this, as well as other limitations highlighted in our discussion.

### Limitations

This review has encountered some limitations. The first is related to the overall moderate to weak methodological quality of the studies analysed. To provide the most comprehensive overview possible of the existing literature, several papers were included in this review that would have not met more stringent methodology-related inclusion / exclusion criteria. This approach, whilst less than optimal, remains in line with standard methodological guidance for systematic reviews (Hannes, 2011). The heterogeneity of the literature retrieved also made both the quality assessment and the analysis of results less than straightforward; the main implication here is that our findings are subject to important caveats, as highlighted throughout this paper.

A second limitation is related to the scope of this review, and the difficulty to conceptually and practically isolate mhGAP-IG implementation from two related, but broader objects of analysis: integration of mental health into community-based services, such as education settings, and task-shifting of specialised tasks to non-specialist personnel. Together, these two dimensions form the essence of the mhGAP project (WHO, 2008a); however, they also represent broader concepts and goals, which this review could not fully explore. A wider consideration of these two aspects in the context of child mental health would provide additional depth to our analysis.

A third limitation is related to the fact that access was limited to published literature available on the main databases. Unpublished documents could not be retrieved or analysed, possibly depriving the review of important insights.

Finally, the review touches upon some critical dimensions – the ontology of childhood, mental health / illness and associated practices reproduced by mhGAP-IG – that our chosen methodology did not allow us to systematically explore. This remains the possible subject of a different, complementary study.

## Conclusions

Despite a common perception that a large body of research evidence exists in support of mhGAP implementation (Akol, 2017; Keynejad, 2018), findings from this review suggest that such evidence, at least as far as child and adolescent mental health is concerned, is very limited. The most strongly corroborated outcomes are related to providers’ knowledge, but significant adjustments in implementation modalities may be needed to consistently translate this knowledge in competencies, practices, and positive health outcomes for children and young people. Some limited evidence of mixed methodological quality indicates that mhGAP-IG, complemented with Parents Skills Training (PTS), may be effective in supporting task shifting to caregivers of children with pervasive developmental disorders and disabilities. Additional studies are currently ongoing, seeking to reinforce this finding.

Our review also suggests that clinical settings have been preferred to community-based settings for mhGAP-IG roll out, and that identification, diagnosis, and treatment of child adolescent mental health disorders have been prioritised over broader mental health prevention and promotion. Limited incorporation of developmental perspectives, and of local systems of knowledge and understanding of childhood and child mental health, was notable in the studies reviewed. Additional research is required to systematically explore this point.

Overall, our findings suggest that the broad and encompassing goals of the mhGAP programme may not be entirely translated in implementation strategies, and therefore in outcomes for children. More attention to the systemic aspects of the implementation process, as indicated in the recently released *mhGAP Operational Manual* (WHO, 2018), in addition to conceptual clarifications around transcultural understandings of mental health and illness, may be required for the programme to reach its objectives.
